# 5-[1-(4-Meth­oxy­phen­yl)-2-nitro­but­yl]-4-phenyl-1,2,3-selenadiazole

**DOI:** 10.1107/S1600536812020752

**Published:** 2012-05-19

**Authors:** P. Sugumar, S. Sankari, P. Manisankar, M. N. Ponnuswamy

**Affiliations:** a Centre of Advanced Study in Crystallography and Biophysics, University of Madras, Guindy Campus, Chennai 600 025, India; b Department of Chemistry, Sri Sarada College for Women (Autonomus), Fairlands, Salem 636 016, India; c Department of Industrial Chemistry, Alagappa University, Karaikudi 630 003, India

## Abstract

In the title compound, C_19_H_19_N_3_O_3_Se, the selenadiazole ring is essentially planar (r.m.s. deviation = 0.001 Å). The heterocyclic ring makes dihedral angles of 50.2 (2) and 76.3 (9)°, respectively, with the meth­oxy­phenyl and phenyl rings.

## Related literature

For general background to selenadiazol derivatives, see: Cuvardic (2003 ▶); El-Bahaie *et al.* (1990 ▶); El-Kashef *et al.* (1986 ▶); Kuroda *et al.* (2001 ▶); Khanna (2005 ▶); Padmavathi *et al.* (2002 ▶); Plano *et al.* (2010 ▶); Stadtman (1991 ▶). For bond-length data, see: Allen *et al.* (1987 ▶).
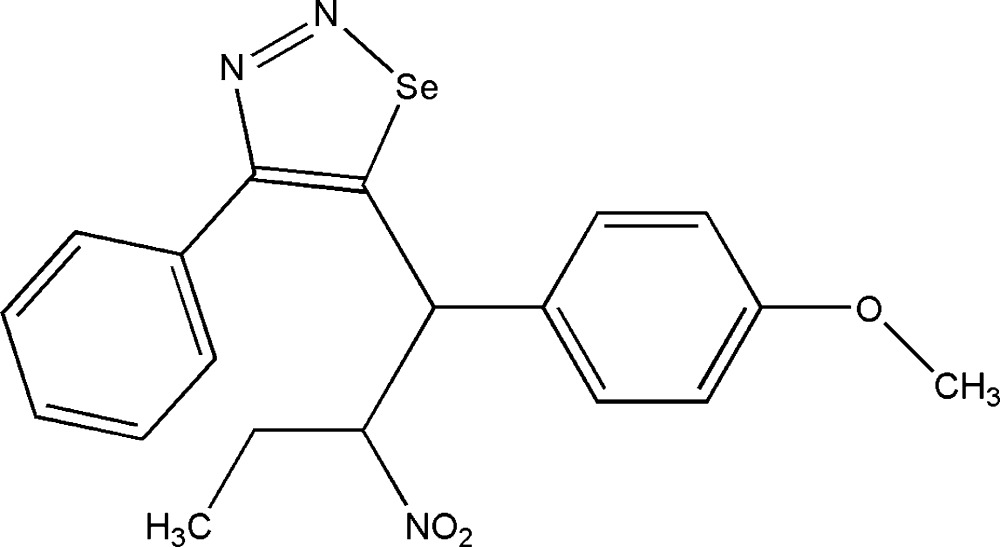


## Experimental

### 

#### Crystal data

C_19_H_19_N_3_O_3_Se
*M* *_r_* = 416.33
Triclinic, 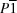

*a* = 8.3072 (5) Å
*b* = 8.5468 (5) Å
*c* = 13.6969 (9) Å
α = 81.293 (3)°
β = 79.670 (3)°
γ = 78.888 (3)°
*V* = 931.88 (10) Å^3^
*Z* = 2
Mo *K*α radiation
μ = 2.04 mm^−1^
*T* = 293 K
0.24 × 0.20 × 0.18 mm

#### Data collection

Bruker SMART APEX CCD detector diffractometer
Absorption correction: multi-scan (*SADABS*; Bruker, 2008 ▶) *T*_min_ = 0.607, *T*_max_ = 0.693
16582 measured reflections
4605 independent reflections
3605 reflections with *I* > 2σ(*I*)
*R*_int_ = 0.024

#### Refinement

*R*[*F* ^2^ > 2σ(*F* ^2^)] = 0.031
*wR*(*F* ^2^) = 0.080
*S* = 1.02
4605 reflections
237 parameters
H-atom parameters constrained
Δρ_max_ = 0.42 e Å^−3^
Δρ_min_ = −0.39 e Å^−3^

### 

Data collection: *APEX2* (Bruker, 2008 ▶); cell refinement: *SAINT* (Bruker, 2008 ▶); data reduction: *SAINT*; program(s) used to solve structure: *SHELXS97* (Sheldrick, 2008 ▶); program(s) used to refine structure: *SHELXL97* (Sheldrick, 2008 ▶); molecular graphics: *ORTEP-3* (Farrugia, 1997 ▶); software used to prepare material for publication: *SHELXL97* and *PLATON* (Spek, 2009 ▶).

## Supplementary Material

Crystal structure: contains datablock(s) global, I. DOI: 10.1107/S1600536812020752/bt5896sup1.cif

Structure factors: contains datablock(s) I. DOI: 10.1107/S1600536812020752/bt5896Isup2.hkl

Supplementary material file. DOI: 10.1107/S1600536812020752/bt5896Isup3.cml

Additional supplementary materials:  crystallographic information; 3D view; checkCIF report

## Figures and Tables

**Fig. 1.**
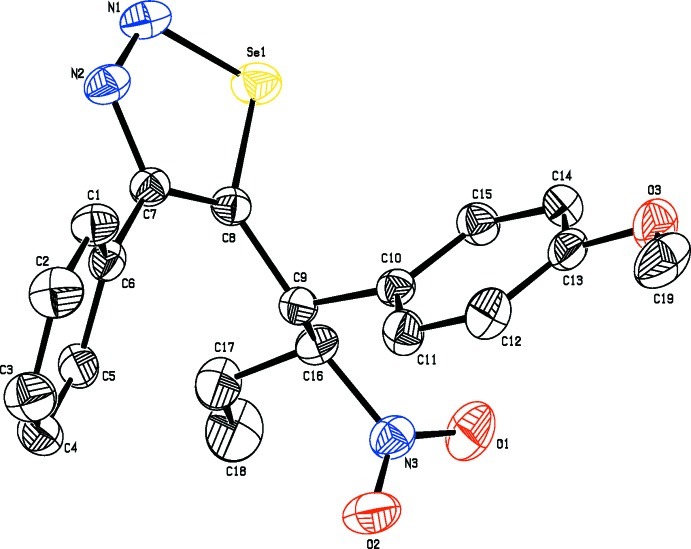
The molecular structure of the title compound, showing the atomic numbering and displacement ellipsoids drawn at 30% probability level. Hydrogen atoms have been omitted for clarity.

**Fig. 2.**
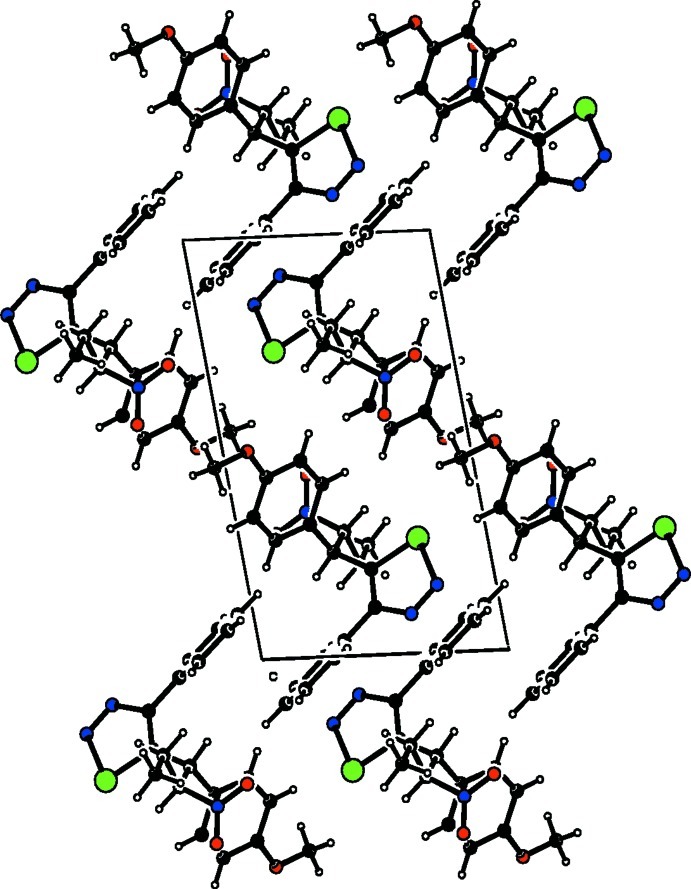
The crystal packing of the molecules viewed down c axis.

## supplementary crystallographic information

### Comment

Selenium containing heterocyclic compounds have gained importance due to their diverse biological, medicinal and synthetic applications. Selenadiazoles, having one selenium and two nitrogen atoms in a five membered ring, are the class of organoselenium compounds utilized in the synthesis of semiconductor nanoparticles (Khanna, 2005). These 1,2,3-selenadiazoles are used as the synthetic intermediates in the preparation of many compounds. In addition, 1,2,3-Selenadiazoles possess biological applications such as anti-fungal (Kuroda *et al.*, 2001), anti-bacterial (El-Kashef *et al.*, 1986), anti-microbial (El-Bahaie *et al.*,1990), anti-cancer (Plano *et al.*, 2010) and insecticidal (Padmavathi *et al.*, 2002) properties. Selenium is an essential microelement, necessary for normal functioning of human and animal organisms. Its deficiency in food and feed causes a number of diseases. In high concentrations, selenium is toxic for humans, animals and plants(Cuvardic, 2003).

Glutathione peroxidases(GPx) are the antioxidant selenoenzymes protecting various organisms from oxidative stress by catalyzing the reduction of hydroperoxides at the expense of glutathione(GSH) (Stadtman, 1991). Owing to the above said important properties of selenium containing compounds, the crystal structure of the title compound is carried out.

The *ORTEP* plot of the molecule is shown in Fig.1. The selenadiazole ring is planar and oriented at an angle of 50.2 (2)° with the attached phenyl ring. The sum of the bond angles around N3 atom in the molecule is 360° indicating *sp^2^* hybridized state. The bond lengths [Se1—N1] 1.879 (2) Å and [Se1—C8] 1.844 (2) Å are comparable with the values reported in the literature (Allen *et al.*, 1987). The selenadiazole ring system and the methoxyphenyl group are oriented at an angle of 76.3 (1)° with respect to each other. In nitro group, the bond lengths [N3—O1] 1.215 (2) Å and [N3—O2] 1.210 (2) Å indicate the typical resonance character. The packing of the molecules viewed down c-axis is shown in Fig.2.

### Experimental

A mixture of 3-(4-methoxyphenyl)-4-nitro-1-phenylhexan-1-one(1 mmol), semicarbazide hydrochloride(2 mmol) and anhydrous sodium acetate(3 mmol) in ethanol(10 ml) was refluxed for 4 h. After completion of the reaction as monitored by TLC, the mixture was poured into ice cold water and the resulting semicarbazone was filtered off. Then, a mixture of semicarbazone(1 mmol) and SeO_2_(2 mmol) in tetrahydrofuran(10 ml) were refluxed on a water bath for 1 h. The selenium deposited on cooling was removed by filtration, and the filtrate was poured into crushed ice, extracted with dichloromethane, and purified by column chromatography using silica gel(60–120 mesh) with 97:3 petroleum ether: ethyl acetate as eluent to give 5-(1-(4-methoxyphenyl) -2-nitrobutyl)-4-phenyl-1,2,3-selenadiazole.

### Refinement

H atoms were positioned geometrically (C—H=0.93–0.98 Å) and allowed to ride on their parent atoms,with *U*_iso_(H) = 1.5*U*_eq_(C) for methyl H 1.2*U*_eq_(C) for other H atoms.

### Crystal data

**Table:**

C_19_H_19_N_3_O_3_Se	*Z* = 2
*M**_r_* = 416.33	*F*(000) = 424
Triclinic, *P*1	*D*_x_ = 1.484 Mg m^−^^3^
Hall symbol: -P 1	Mo *K*α radiation, λ = 0.71073 Å
*a* = 8.3072 (5) Å	Cell parameters from 3605 reflections
*b* = 8.5468 (5) Å	θ = 1.5–28.3°
*c* = 13.6969 (9) Å	µ = 2.04 mm^−^^1^
α = 81.293 (3)°	*T* = 293 K
β = 79.670 (3)°	Block, colourless
γ = 78.888 (3)°	0.24 × 0.20 × 0.18 mm
*V* = 931.88 (10) Å^3^	

### Data collection

**Table:**

Bruker SMART APEX CCD detector diffractometer	4605 independent reflections
Radiation source: fine-focus sealed tube	3605 reflections with *I* > 2σ(*I*)
Graphite monochromator	*R*_int_ = 0.024
ω scans	θ_max_ = 28.3°, θ_min_ = 1.5°
Absorption correction: multi-scan (*SADABS*; Bruker, 2008)	*h* = −11→11
*T*_min_ = 0.607, *T*_max_ = 0.693	*k* = −11→11
16582 measured reflections	*l* = −17→18

### Refinement

**Table:**

Refinement on *F*^2^	Primary atom site location: structure-invariant direct methods
Least-squares matrix: full	Secondary atom site location: difference Fourier map
*R*[*F*^2^ > 2σ(*F*^2^)] = 0.031	Hydrogen site location: inferred from neighbouring sites
*wR*(*F*^2^) = 0.080	H-atom parameters constrained
*S* = 1.02	*w* = 1/[σ^2^(*F*_o_^2^) + (0.0368*P*)^2^ + 0.2368*P*] where *P* = (*F*_o_^2^ + 2*F*_c_^2^)/3
4605 reflections	(Δ/σ)_max_ = 0.021
237 parameters	Δρ_max_ = 0.42 e Å^−^^3^
0 restraints	Δρ_min_ = −0.39 e Å^−^^3^

### Special details

**Table:**

Geometry. All e.s.d.'s (except the e.s.d. in the dihedral angle between two l.s. planes) are estimated using the full covariance matrix. The cell e.s.d.'s are taken into account individually in the estimation of e.s.d.'s in distances, angles and torsion angles; correlations between e.s.d.'s in cell parameters are only used when they are defined by crystal symmetry. An approximate (isotropic) treatment of cell e.s.d.'s is used for estimating e.s.d.'s involving l.s. planes.
Refinement. Refinement of *F*^2^ against ALL reflections. The weighted *R*-factor *wR* and goodness of fit *S* are based on *F*^2^, conventional *R*-factors *R* are based on *F*, with *F* set to zero for negative *F*^2^. The threshold expression of *F*^2^ > σ(*F*^2^) is used only for calculating *R*-factors(gt) *etc*. and is not relevant to the choice of reflections for refinement. *R*-factors based on *F*^2^ are statistically about twice as large as those based on *F*, and *R*- factors based on ALL data will be even larger.

### Fractional atomic coordinates and isotropic or equivalent isotropic displacement parameters (Å^2^)

**Table:**

	*x*	*y*	*z*	*U*_iso_*/*U*_eq_	
C1	0.7080 (3)	0.1143 (2)	−0.00387 (15)	0.0532 (5)	
H1	0.6485	0.0305	0.0178	0.064*	
C2	0.8475 (3)	0.0937 (3)	−0.07483 (17)	0.0673 (6)	
H2	0.8810	−0.0037	−0.1011	0.081*	
C3	0.9378 (3)	0.2151 (3)	−0.10723 (17)	0.0697 (6)	
H3	1.0337	0.1992	−0.1540	0.084*	
C4	0.8857 (3)	0.3608 (3)	−0.07019 (15)	0.0619 (5)	
H4	0.9453	0.4442	−0.0930	0.074*	
C5	0.7454 (3)	0.3830 (2)	0.00070 (13)	0.0502 (4)	
H5	0.7108	0.4816	0.0253	0.060*	
C6	0.6551 (2)	0.2590 (2)	0.03568 (12)	0.0427 (4)	
C7	0.5064 (2)	0.27772 (19)	0.11255 (13)	0.0420 (4)	
C8	0.4926 (2)	0.33050 (19)	0.20352 (13)	0.0414 (4)	
C9	0.6313 (2)	0.37263 (19)	0.24707 (12)	0.0403 (4)	
H9	0.7190	0.3932	0.1909	0.048*	
C10	0.7054 (2)	0.23002 (19)	0.31467 (12)	0.0404 (4)	
C11	0.8557 (2)	0.1404 (2)	0.28056 (14)	0.0514 (4)	
H11	0.9122	0.1725	0.2180	0.062*	
C12	0.9254 (3)	0.0033 (3)	0.33688 (16)	0.0582 (5)	
H12	1.0266	−0.0558	0.3118	0.070*	
C13	0.8438 (3)	−0.0443 (2)	0.42993 (15)	0.0516 (5)	
C14	0.6949 (3)	0.0457 (2)	0.46637 (14)	0.0557 (5)	
H14	0.6406	0.0151	0.5299	0.067*	
C15	0.6259 (2)	0.1806 (2)	0.40976 (14)	0.0500 (4)	
H15	0.5249	0.2395	0.4353	0.060*	
C16	0.5739 (2)	0.5295 (2)	0.29509 (14)	0.0461 (4)	
H16	0.4754	0.5191	0.3454	0.055*	
C17	0.5340 (3)	0.6751 (2)	0.21955 (17)	0.0648 (6)	
H17A	0.4426	0.6606	0.1885	0.078*	
H17B	0.6296	0.6801	0.1676	0.078*	
C18	0.4881 (4)	0.8345 (3)	0.2628 (2)	0.0881 (8)	
H18A	0.3973	0.8291	0.3168	0.132*	
H18B	0.4559	0.9189	0.2117	0.132*	
H18C	0.5821	0.8560	0.2871	0.132*	
C19	1.0446 (4)	−0.2802 (3)	0.4575 (2)	0.0879 (9)	
H19A	1.0289	−0.3208	0.3989	0.132*	
H19B	1.0674	−0.3681	0.5085	0.132*	
H19C	1.1362	−0.2227	0.4413	0.132*	
N1	0.2397 (2)	0.2408 (2)	0.16056 (14)	0.0591 (4)	
N2	0.3652 (2)	0.23047 (18)	0.09389 (13)	0.0521 (4)	
N3	0.7123 (2)	0.55482 (18)	0.34525 (13)	0.0539 (4)	
O1	0.6804 (2)	0.5740 (2)	0.43322 (12)	0.0790 (5)	
O2	0.8482 (2)	0.5549 (2)	0.29537 (14)	0.0783 (5)	
O3	0.8996 (2)	−0.17608 (19)	0.49253 (13)	0.0751 (5)	
Se1	0.28223 (2)	0.31972 (3)	0.272539 (16)	0.06042 (9)	

### Atomic displacement parameters (Å^2^)

**Table:**

	*U*^11^	*U*^22^	*U*^33^	*U*^12^	*U*^13^	*U*^23^
C1	0.0558 (11)	0.0459 (10)	0.0621 (11)	−0.0131 (9)	−0.0071 (9)	−0.0166 (8)
C2	0.0680 (14)	0.0640 (13)	0.0723 (14)	−0.0091 (11)	0.0006 (11)	−0.0321 (11)
C3	0.0630 (13)	0.0850 (16)	0.0616 (13)	−0.0177 (12)	0.0069 (10)	−0.0240 (11)
C4	0.0698 (14)	0.0678 (13)	0.0506 (11)	−0.0279 (11)	0.0000 (10)	−0.0061 (9)
C5	0.0643 (12)	0.0443 (9)	0.0439 (9)	−0.0139 (9)	−0.0083 (8)	−0.0061 (7)
C6	0.0469 (10)	0.0421 (9)	0.0420 (9)	−0.0074 (8)	−0.0123 (7)	−0.0075 (7)
C7	0.0456 (9)	0.0330 (8)	0.0498 (9)	−0.0083 (7)	−0.0110 (8)	−0.0059 (7)
C8	0.0410 (9)	0.0364 (8)	0.0463 (9)	−0.0066 (7)	−0.0057 (7)	−0.0048 (7)
C9	0.0416 (9)	0.0411 (8)	0.0390 (8)	−0.0101 (7)	−0.0025 (7)	−0.0083 (7)
C10	0.0412 (9)	0.0425 (9)	0.0402 (8)	−0.0110 (7)	−0.0049 (7)	−0.0104 (7)
C11	0.0456 (10)	0.0617 (11)	0.0437 (9)	−0.0043 (9)	−0.0015 (8)	−0.0088 (8)
C12	0.0499 (11)	0.0595 (12)	0.0630 (12)	0.0062 (9)	−0.0124 (9)	−0.0161 (10)
C13	0.0604 (12)	0.0458 (10)	0.0563 (11)	−0.0158 (9)	−0.0253 (9)	−0.0028 (8)
C14	0.0620 (12)	0.0632 (12)	0.0448 (10)	−0.0249 (10)	−0.0085 (9)	0.0025 (9)
C15	0.0458 (10)	0.0560 (11)	0.0463 (10)	−0.0097 (8)	0.0013 (8)	−0.0091 (8)
C16	0.0446 (10)	0.0443 (9)	0.0524 (10)	−0.0090 (8)	−0.0085 (8)	−0.0119 (8)
C17	0.0813 (16)	0.0468 (11)	0.0701 (14)	−0.0062 (10)	−0.0249 (12)	−0.0093 (10)
C18	0.113 (2)	0.0459 (12)	0.107 (2)	0.0026 (13)	−0.0318 (18)	−0.0174 (12)
C19	0.0873 (19)	0.0537 (13)	0.134 (2)	−0.0043 (13)	−0.0648 (18)	0.0000 (14)
N1	0.0493 (9)	0.0564 (10)	0.0787 (12)	−0.0193 (8)	−0.0129 (9)	−0.0136 (8)
N2	0.0518 (9)	0.0449 (8)	0.0661 (10)	−0.0138 (7)	−0.0157 (8)	−0.0117 (7)
N3	0.0590 (10)	0.0465 (8)	0.0613 (10)	−0.0122 (8)	−0.0142 (8)	−0.0126 (7)
O1	0.0894 (12)	0.0929 (12)	0.0644 (10)	−0.0116 (10)	−0.0205 (9)	−0.0349 (9)
O2	0.0577 (9)	0.0962 (12)	0.0904 (12)	−0.0340 (9)	−0.0057 (9)	−0.0215 (9)
O3	0.0899 (12)	0.0609 (9)	0.0800 (11)	−0.0170 (9)	−0.0390 (9)	0.0097 (8)
Se1	0.04506 (12)	0.07248 (16)	0.06460 (15)	−0.01676 (10)	0.00230 (9)	−0.01524 (10)

### Geometric parameters (Å, º)

**Table:**

C1—C2	1.374 (3)	C12—H12	0.9300
C1—C6	1.387 (2)	C13—O3	1.366 (2)
C1—H1	0.9300	C13—C14	1.378 (3)
C2—C3	1.370 (3)	C14—C15	1.375 (3)
C2—H2	0.9300	C14—H14	0.9300
C3—C4	1.378 (3)	C15—H15	0.9300
C3—H3	0.9300	C16—N3	1.505 (2)
C4—C5	1.379 (3)	C16—C17	1.517 (3)
C4—H4	0.9300	C16—H16	0.9800
C5—C6	1.393 (2)	C17—C18	1.521 (3)
C5—H5	0.9300	C17—H17A	0.9700
C6—C7	1.473 (2)	C17—H17B	0.9700
C7—C8	1.367 (2)	C18—H18A	0.9600
C7—N2	1.388 (2)	C18—H18B	0.9600
C8—C9	1.512 (2)	C18—H18C	0.9600
C8—Se1	1.8438 (17)	C19—O3	1.407 (3)
C9—C10	1.515 (2)	C19—H19A	0.9600
C9—C16	1.538 (2)	C19—H19B	0.9600
C9—H9	0.9800	C19—H19C	0.9600
C10—C11	1.377 (2)	N1—N2	1.255 (2)
C10—C15	1.394 (2)	N1—Se1	1.8792 (18)
C11—C12	1.388 (3)	N3—O2	1.210 (2)
C11—H11	0.9300	N3—O1	1.215 (2)
C12—C13	1.374 (3)		
			
C2—C1—C6	120.59 (18)	O3—C13—C14	115.45 (18)
C2—C1—H1	119.7	C12—C13—C14	119.47 (18)
C6—C1—H1	119.7	C15—C14—C13	120.66 (18)
C3—C2—C1	120.67 (19)	C15—C14—H14	119.7
C3—C2—H2	119.7	C13—C14—H14	119.7
C1—C2—H2	119.7	C14—C15—C10	120.88 (18)
C2—C3—C4	119.7 (2)	C14—C15—H15	119.6
C2—C3—H3	120.2	C10—C15—H15	119.6
C4—C3—H3	120.2	N3—C16—C17	108.37 (15)
C3—C4—C5	120.12 (19)	N3—C16—C9	107.81 (14)
C3—C4—H4	119.9	C17—C16—C9	112.92 (15)
C5—C4—H4	119.9	N3—C16—H16	109.2
C4—C5—C6	120.54 (18)	C17—C16—H16	109.2
C4—C5—H5	119.7	C9—C16—H16	109.2
C6—C5—H5	119.7	C16—C17—C18	114.67 (19)
C1—C6—C5	118.39 (17)	C16—C17—H17A	108.6
C1—C6—C7	119.66 (16)	C18—C17—H17A	108.6
C5—C6—C7	121.95 (15)	C16—C17—H17B	108.6
C8—C7—N2	115.27 (16)	C18—C17—H17B	108.6
C8—C7—C6	127.29 (16)	H17A—C17—H17B	107.6
N2—C7—C6	117.37 (15)	C17—C18—H18A	109.5
C7—C8—C9	126.18 (15)	C17—C18—H18B	109.5
C7—C8—Se1	109.09 (13)	H18A—C18—H18B	109.5
C9—C8—Se1	124.37 (12)	C17—C18—H18C	109.5
C8—C9—C10	110.23 (13)	H18A—C18—H18C	109.5
C8—C9—C16	110.96 (14)	H18B—C18—H18C	109.5
C10—C9—C16	115.08 (14)	O3—C19—H19A	109.5
C8—C9—H9	106.7	O3—C19—H19B	109.5
C10—C9—H9	106.7	H19A—C19—H19B	109.5
C16—C9—H9	106.7	O3—C19—H19C	109.5
C11—C10—C15	117.54 (17)	H19A—C19—H19C	109.5
C11—C10—C9	119.27 (15)	H19B—C19—H19C	109.5
C15—C10—C9	123.16 (16)	N2—N1—Se1	111.05 (12)
C10—C11—C12	121.92 (17)	N1—N2—C7	117.76 (16)
C10—C11—H11	119.0	O2—N3—O1	124.24 (19)
C12—C11—H11	119.0	O2—N3—C16	117.87 (17)
C13—C12—C11	119.51 (18)	O1—N3—C16	117.89 (17)
C13—C12—H12	120.2	C13—O3—C19	118.6 (2)
C11—C12—H12	120.2	C8—Se1—N1	86.83 (7)
O3—C13—C12	125.1 (2)		
			
C6—C1—C2—C3	0.5 (4)	C10—C11—C12—C13	−0.7 (3)
C1—C2—C3—C4	−1.7 (4)	C11—C12—C13—O3	−179.72 (18)
C2—C3—C4—C5	1.4 (4)	C11—C12—C13—C14	−0.7 (3)
C3—C4—C5—C6	0.1 (3)	O3—C13—C14—C15	−179.59 (17)
C2—C1—C6—C5	0.9 (3)	C12—C13—C14—C15	1.3 (3)
C2—C1—C6—C7	−179.2 (2)	C13—C14—C15—C10	−0.5 (3)
C4—C5—C6—C1	−1.2 (3)	C11—C10—C15—C14	−0.9 (3)
C4—C5—C6—C7	178.94 (19)	C9—C10—C15—C14	176.95 (16)
C1—C6—C7—C8	128.2 (2)	C8—C9—C16—N3	174.57 (14)
C5—C6—C7—C8	−52.0 (3)	C10—C9—C16—N3	48.55 (19)
C1—C6—C7—N2	−48.7 (2)	C8—C9—C16—C17	−65.7 (2)
C5—C6—C7—N2	131.17 (18)	C10—C9—C16—C17	168.24 (16)
N2—C7—C8—C9	173.33 (15)	N3—C16—C17—C18	−56.6 (3)
C6—C7—C8—C9	−3.6 (3)	C9—C16—C17—C18	−176.0 (2)
N2—C7—C8—Se1	0.06 (19)	Se1—N1—N2—C7	−0.5 (2)
C6—C7—C8—Se1	−176.86 (14)	C8—C7—N2—N1	0.3 (2)
C7—C8—C9—C10	−95.23 (19)	C6—C7—N2—N1	177.56 (16)
Se1—C8—C9—C10	77.06 (17)	C17—C16—N3—O2	−68.0 (2)
C7—C8—C9—C16	136.09 (17)	C9—C16—N3—O2	54.5 (2)
Se1—C8—C9—C16	−51.62 (19)	C17—C16—N3—O1	112.0 (2)
C8—C9—C10—C11	103.29 (18)	C9—C16—N3—O1	−125.52 (18)
C16—C9—C10—C11	−130.31 (17)	C12—C13—O3—C19	−5.6 (3)
C8—C9—C10—C15	−74.5 (2)	C14—C13—O3—C19	175.42 (18)
C16—C9—C10—C15	51.9 (2)	C7—C8—Se1—N1	−0.26 (13)
C15—C10—C11—C12	1.5 (3)	C9—C8—Se1—N1	−173.68 (15)
C9—C10—C11—C12	−176.43 (17)	N2—N1—Se1—C8	0.44 (14)
